# DNA sequence polymorphisms within the bovine guanine nucleotide-binding protein Gs subunit alpha (Gsα)-encoding (*GNAS*) genomic imprinting domain are associated with performance traits

**DOI:** 10.1186/1471-2156-12-4

**Published:** 2011-01-07

**Authors:** Klaudia M Sikora, David A Magee, Erik W Berkowicz, Donagh P Berry, Dawn J Howard, Michael P Mullen, Ross D Evans, David E MacHugh, Charles Spillane

**Affiliations:** 1 Genetics and Biotechnology Laboratory, Department of Biochemistry, University College Cork, Cork, Ireland; 2 Genetics and Biotechnology Laboratory, Centre for Chromosome Biology, C306 Aras de Brun, National University of Ireland Galway, Galway, Ireland; 3 Animal Genomics Laboratory, UCD School of Agriculture, Food Science and Veterinary Medicine, University College Dublin, Belfield, Dublin 4, Ireland; 4 Animal and Bioscience Research Department, Animal and Grassland Research and Innovation Centre, Teagasc, Fermoy, Co. Cork, Ireland; 5 Animal and Bioscience Research Department, Animal and Grassland Research and Innovation Centre, Teagasc, Mellows Campus, Athenry, Co. Galway, Ireland; 6 Irish Cattle Breeding Federation, Highfield House, Bandon, Co. Cork, Ireland; 7 UCD Conway Institute of Biomolecular and Biomedical Research, University College Dublin, Dublin 4, Ireland

## Abstract

**Background:**

Genes which are epigenetically regulated via genomic imprinting can be potential targets for artificial selection during animal breeding. Indeed, imprinted loci have been shown to underlie some important quantitative traits in domestic mammals, most notably muscle mass and fat deposition. In this candidate gene study, we have identified novel associations between six validated single nucleotide polymorphisms (SNPs) spanning a 97.6 kb region within the bovine guanine nucleotide-binding protein Gs subunit alpha gene (*GNAS*) domain on bovine chromosome 13 and genetic merit for a range of performance traits in 848 progeny-tested Holstein-Friesian sires. The mammalian *GNAS *domain consists of a number of reciprocally-imprinted, alternatively-spliced genes which can play a major role in growth, development and disease in mice and humans. Based on the current annotation of the bovine *GNAS *domain, four of the SNPs analysed (*rs43101491*, *rs43101493*, *rs43101485 *and *rs43101486*) were located upstream of the *GNAS *gene, while one SNP (*rs41694646*) was located in the second intron of the *GNAS *gene. The final SNP (*rs41694656*) was located in the first exon of transcripts encoding the putative bovine neuroendocrine-specific protein NESP55, resulting in an aspartic acid-to-asparagine amino acid substitution at amino acid position 192.

**Results:**

SNP genotype-phenotype association analyses indicate that the single intronic *GNAS *SNP (*rs41694646*) is associated (*P *≤ 0.05) with a range of performance traits including milk yield, milk protein yield, the content of fat and protein in milk, culled cow carcass weight and progeny carcass conformation, measures of animal body size, direct calving difficulty (*i.e*. difficulty in calving due to the size of the calf) and gestation length. Association (*P *≤ 0.01) with direct calving difficulty (*i.e*. due to calf size) and maternal calving difficulty (*i.e*. due to the maternal pelvic width size) was also observed at the *rs43101491 *SNP. Following adjustment for multiple-testing, significant association (*q *≤ 0.05) remained between the *rs41694646 *SNP and four traits (animal stature, body depth, direct calving difficulty and milk yield) only. Notably, the single SNP in the bovine *NESP55 *gene (*rs41694656*) was associated (*P *≤ 0.01) with somatic cell count--an often-cited indicator of resistance to mastitis and overall health status of the mammary system--and previous studies have demonstrated that the chromosomal region to where the *GNAS *domain maps underlies an important quantitative trait locus for this trait. This association, however, was not significant after adjustment for multiple testing. The three remaining SNPs assayed were not associated with any of the performance traits analysed in this study. Analysis of all pairwise linkage disequilibrium (*r*^2^) values suggests that most allele substitution effects for the assayed SNPs observed are independent. Finally, the polymorphic coding SNP in the putative bovine *NESP55 *gene was used to test the imprinting status of this gene across a range of foetal bovine tissues.

**Conclusions:**

Previous studies in other mammalian species have shown that DNA sequence variation within the imprinted *GNAS *gene cluster contributes to several physiological and metabolic disorders, including obesity in humans and mice. Similarly, the results presented here indicate an important role for the imprinted *GNAS *cluster in underlying complex performance traits in cattle such as animal growth, calving, fertility and health. These findings suggest that *GNAS *domain-associated polymorphisms may serve as important genetic markers for future livestock breeding programs and support previous studies that candidate imprinted loci may act as molecular targets for the genetic improvement of agricultural populations. In addition, we present new evidence that the bovine *NESP55 *gene is epigenetically regulated as a maternally expressed imprinted gene in placental and intestinal tissues from 8-10 week old bovine foetuses.

## Background

Genomic imprinting is a form of epigenetic regulation which results in the complete or preferential monoallelic expression of approximately 100 mammalian autosomal genes in a parent-of-origin dependent manner [1-6]. Genes subject to this form of epigenetic control have been shown to play major roles in regulation of mammalian postnatal growth, development, and metabolism. Furthermore, perturbations of the imprinting status of these genes (*i.e*. loss of imprinting) can result in serious physiological impairments (such as those associated with Angleman syndrome and Beckwith-Wiedemann syndrome in humans), lethality and susceptibility to diseases such as cancer [7-9].

Imprinted genes are organised into clusters or domains within the mammalian genome, in which both paternally-expressed and maternally-expressed genes (*i.e*. reciprocally-imprinted genes, including both protein-coding and regulatory non-coding RNAs genes) occur at a higher density than other regions of the genome [10,11]. One such cluster of imprinted genes is the mammalian *GNAS *domain which consists of a number of imprinted genes which display complex transcriptional and epigenetic regulation [12,13]. In humans, the *GNAS *domain spans ~70 kilobases (kb) on chromosome 20 and displays similar gene organisation and imprinting patterns to the orthologous *Gnas *domain on murine chromosome 2 [14]. An integral member of this domain is the *GNAS *gene which encodes the alpha-stimulatory subunit of the trimeric guanine nucleotide-binding (or G-protein, G_S_α). G-proteins are involved in both the coupling of many hormone and neurotransmitter receptor proteins to adenylate cyclase and also the production of cyclic adenosine monophosphate (cAMP) for downstream cellular signal transduction pathways [15]. The human *GNAS *gene, transcribed from the G_S_α promoter, consists of 13 exons (the mouse gene model contains 12 exons) and is predominantly biallelically expressed, except in a subset of cells and tissues--including proximal renal tubule cells, thyroid and anterior pituitary glands and ovaries--where preferential expression of the maternally inherited allele is observed [16]. Maternal-specific expression has also been documented in neonatal adipose tissues, while the reporting of biallelic expression in adult human adipose tissue suggests that imprinting of *GNAS *is both tissue- and developmental-stage specific [16].

Other mRNAs produced within the mammalian *GNAS *domain include the reciprocally-imprinted *GNASxl *and *NESP55 *transcripts, both of which are generated through the use of alternative promoters and first exons that splice to the common exon 2 of the *GNAS *gene [13]. The paternally-expressed *GNASxl *transcript encodes the G_S_α isoform, XLα_S _('extralarge G_S_α'), and is synthesised via alternative splicing of the XLα_S _exon (located ~32.5 kb upstream of the G_S_α promoter) to exons 2-13 of the *GNAS *gene [17]. The maternally-expressed neuroendocrine-specific *NESP55 *transcript is produced by the splicing of the *NESP *exon (located ~45.7 kb upstream of the G_S_α promoter) and *GNAS *exons 2-12. *NESP55 *transcripts encode a 55 kDa neuroendocrine secretory, chromogranin-like protein of unknown function [18]. The entire coding region of the NESP55 protein is located within the *NESP *exon while *GNAS *exons 2-13 constitute the 3'UTR of *NESP55 *transcripts [19].

Recently, a number of studies have highlighted the relationship between the *GNAS *imprinting domain and the development of disease in both mice and humans. For example, genetic perturbations within the *GNAS *domain, such as point mutations and duplication/deletion of maternally- or paternally-inherited alleles, can result in physiological dysfunction, such as reduced body size, hypermetabolism, obesity, mental retardation or neonatal lethality [16,20-24]. Such findings support a major functional role for the *GNAS *domain in regulating mammalian growth and maturation.

Based on the known physiological role of the imprinted *GNAS *domain in regulating mammalian growth and development, we have adopted a candidate gene strategy by assessing associations between six bovine *GNAS *domain SNPs and genetic merit for a range of economically-important performance traits in 848 progeny-tested Holstein-Friesian sires. The candidate gene approach uses variation in genes of known biological function relevant to the trait(s) of interest to investigate genotype-phenotype associations, and is regarded as a viable alternative to whole genome scans for the detection and characterisation of quantitative trait loci (QTL) for complex performance traits [25-27]. Furthermore, in support of our approach, recent investigations have shown that known or candidate imprinted loci (based on the imprinting status of orthologous genes from other mammalian species) can underlie important QTL for complex performance traits in livestock, including animal growth and development [28-36], fat deposition [37], meat traits [38], milk traits [39,40] and fertility traits [41]. In addition, other recent studies have considered the effect of genetic imprinting on quantitative traits in managed populations [42,43].

In cattle, the *GNAS *domain is located on *Bos taurus *chromosome 13 (BTA13). While there is currently no definitive evidence demonstrating that the bovine *GNAS *locus is imprinted, studies have confirmed that this gene is maternally expressed in parthenogenetic bovine embryos [44,45]. However, the appreciable evolutionary conservation of imprinting domains across mammalian species, including humans, mouse, sheep, pigs and cattle, suggests that the bovine *GNAS *locus may also be under similar epigenetic regulation [46-50]. Indeed, preferential maternal expression of the bovine *NESP55 *gene has been previously reported by Khatib [51] in foetal tissue samples. In the current study, while we could not test for imprinting of the *GNAS *gene (due to lack of expressed, coding SNPs), we could validate and extend the preferential maternal expression of the bovine *NESP55 *gene to a wider range of tissues at earlier stages of development to tissues screened in previous studies [51].

## Results

### *NESP55 *is epigenetically regulated as a maternally expressed imprinted gene in 8-10 week old bovine foetal tissues

To determine the imprinting status of the bovine *GNAS *and *NESP55 *genes, exonic and UTR SNPs reported in Build 4.0 of the *B. taurus *genome assembly within the ENSEMBL database http://www.ensembl.org were catalogued and subsequently validated via direct bi-directional sequencing of high-fidelity polymerase chain reaction (PCR) amplicons from genomic DNA using a panel of foetal and dam samples. In this study each of the three predicted gene models for *GNAS *in the cattle genome were considered [ENSEMBL database transcript IDs ENSBTAT00000002746 encodes the bovine *GNAS *transcript, ENSBTAT00000023246 encodes the alternatively spliced transcript termed *GNAS2_BOVIN *and ENSBTAT00000023234 encodes a novel alternatively spliced *GNAS *transcript]. While the ENSEMBL database reports one SNP (*rs41255672*) in the 3'UTR of *GNAS*, our sequencing of PCR amplicons generated from genomic DNA isolated from 10 different animals (using amplicons spanning exons 7 to 14 of the bovine *GNAS *locus) failed to detect a heterozygous SNPs that would allow for a test of *GNAS *imprinting in tissue samples derived from each animal (results not shown).

**Figure 1:**
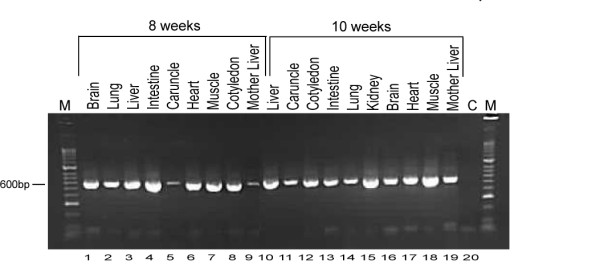
**Reverse-transcriptase (RT)-PCR of *NESP55 *expression in foetal and adult tissues**. RT-PCR primers were located in the *NESP55 *exon (forward primer) and *GNAS *exon 6 (reverse primer). Lanes 1-8: RT-PCR product of tissues from 8 weeks old foetus; lane 9 and 19: RT-PCR product from liver from the dam of 8 and 10 weeks old foetus, respectively; lanes 10-18: RT-PCR product of tissues from 10 week old foetus. Amplified fragments were confirmed by sequencing to be *NESP55 *transcript. C - PCR negative control; M - HyperLadder II Marker (Bioline Ltd, London, UK).

In contrast to the monomorphism of the *rs41255672 *SNP, a coding SNP within *NESP55 *(*rs41694656*) was polymorphic in four foetuses ranging in age from 8 to 10 weeks old. Reverse-transcription PCR (RT-PCR) analysis of RNA samples derived from a wide range of tissues for these foetuses indicated that the *NESP55 *transcript was expressed in brain, heart, intestine, liver, lung, muscle and placental (caruncle and cotyledon) tissues in both 8 and 10 week old foetuses (Figure 1). It has previously been demonstrated that the *NESP55 *exon is spliced to exon 2 of *GNAS *in the cattle samples tested, which is similar to humans and mice [51]. To confirm such splicing for the samples used in this study, the complementary DNA (cDNA) of the *NESP55 *transcripts which spanned the predicted *NESP55 *exon-*GNAS *exon 2 splice junction was sequenced and also confirmed that the *NESP55 *exon splices to *GNAS *exon 2 in cattle.

**Figure 2:**
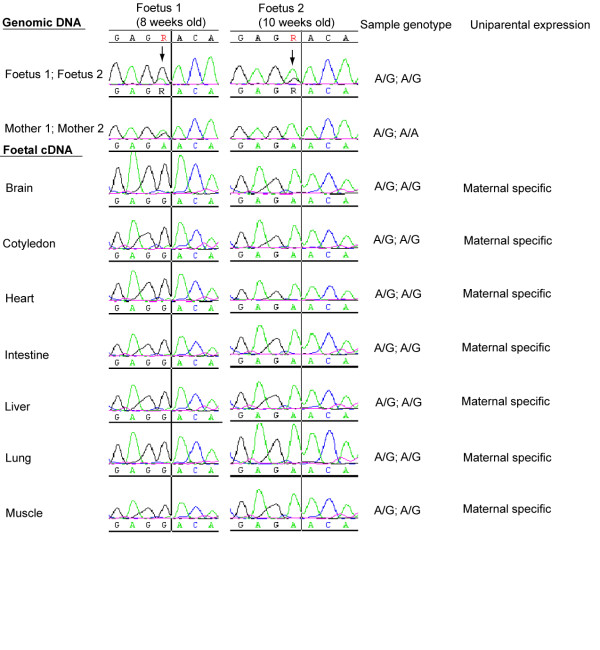
**Sequencing traces of *NESP55 *gene for genomic DNA and cDNA from both maternal and foetal tissues**. The genomic DNA from the mother of each foetus allowed genotyping of the maternal genotype, while the genomic DNA from each foetus allowed genotyping of the each foetus. Arrows indicate the exonic SNP, denoted R (A/G), which was used for both DNA genotyping and to analyse allele-specific expression status of *NESP55 *transcripts (cDNA) across tissues from 8 and 10 weeks old foetuses. Column number 4 presents the genotypes for all samples/animals tested. Column 5 indicates whether monoallelic (*i.e*. uniparental) expression was detected.

While Khatib [51] has demonstrated that *NESP55 *is maternally expressed in a range of foetal tissues from 10-13 weeks old, the imprinting status of *NESP55 *at earlier foetal stages (*e.g*. 8 weeks) remains unknown. Using the coding SNP (*rs41694656*) within *NESP55 *and DNA sequence traces for genomic DNA and cDNA template samples, the imprinting status of *NESP55 *was analysed in the current study in the four A/G heterozygous foetal samples which were either 8 or 10 weeks old (Figure 2). As the dam of foetus 2 was homozygous A/A at this SNP, and expression of only the A allele was observed in all of the heterozygous A/G foetal offspring tissues tested, the results suggest that *NESP55 *is a maternally expressed imprinted gene in seven foetal tissues, all of which are at an earlier developmental stage to those previously analysed by Khatib [51]. Furthermore, our results extend previous demonstrations of *NESP55 *imprinting by demonstrating here that *NESP55 *is also a maternally expressed imprinted gene in intestinal and placental tissues (cotyledon) from both 8 and 10 week old foetal samples (Figure 2).

### Allele and genotype frequencies for the six *GNAS *domain SNPs analysed in 848 progeny-tested Irish Holstein-Friesian sires

**Figure 3:**
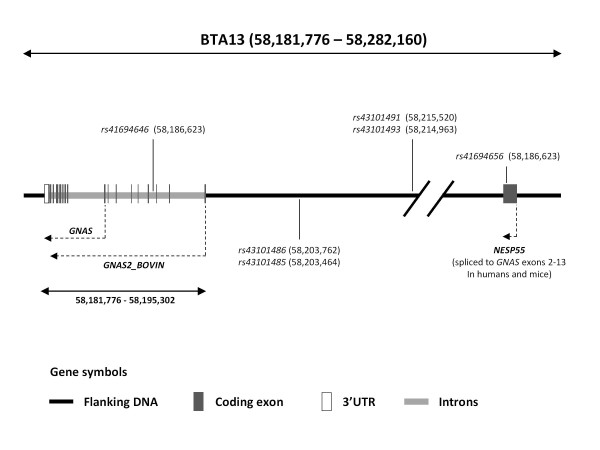
**Annotation of the *GNAS *imprinting region on BTA13 as per build 4.0 of the *B. taurus *genome sequence**. The physical location of each SNP used in this study is indicated in relation to contig BTA13. The SNPs analysed in this study are listed as per their dbSNP accession number http://www.ncbi.nlm.nih.gov/projects/SNP. Two of the reported alternatively spliced *GNAS *transcripts (ENSBTAT00000002746 [*GNAS*] and ENSBTAT00000023246 [G*NAS2_BOVIN*]) are shown here. A third novel *GNAS *alternatively spliced transcript reported in the ENSEMBL database (ENSBTAT00000023234) is not shown. The direction of transcription is denoted by the hashed lines containing arrowheads. The location of the putative bovine *NESP55 *first exon is shown and was determined via alignment of the bovine *NESP55 *mRNA sequence with build 4.0 of the *B. taurus *genome. In humans and mice transcripts from this exon are spliced to exons 2-13 of the *GNAS *gene.

To assess if DNA sequence variation within the bovine *GNAS *domain on BTA13 is associated with bovine performance traits, six SNPs (all previously reported in the ENSEMBL database and validated by us in a previous study [52]) were genotyped in 848 progeny-tested artificial insemination Irish Holstein-Friesian sires. Summary statistics, including genotype and allele frequencies together with deviations from Hardy-Weinberg equilibrium (HWE), for each of the six validated *GNAS *domain SNPs, are presented in Table 1. SNPs were coded as per their unique dbSNP database accession numbers [53], while the genomic co-ordinates for each SNP were taken from the dbSNP database and are based on Build 4.0 of the *B. taurus *genome assembly (ENSEMBL release 60). The position of each SNP relative to the bovine *GNAS *and *NESP55 *genes (*i.e*. upstream/intronic/exonic) was based on the currently annotated transcriptional units contained within the *B. taurus GNAS *domain. The position of the six assayed SNPs within the *GNAS *domain is depicted schematically in Figure 3. *r*^2 ^[54] and *D*' [55] values of linkage disequilibrium (LD) between all pairwise combinations of *GNAS *domain SNPs are presented in Additional file 1. For all assayed SNPs, minor allele frequencies (MAFs) ranged between 0.09 (*rs43101491*) and 0.32 (*rs41694646*). Heterozygosity (*i.e*. the observed number of heterozygous individuals at all six analysed loci) for the six SNPs ranged between 0.17 (*rs43101491*) and 0.45 (*rs41694646*). None of the SNPs assessed demonstrated significant deviation from HWE.

**Table 1:** Summary statistics for the GNAS domain SNPs analysed in this study across 848 Holstein-Friesian sires

SNP	Open reading frame (ORF) model position of SNP	SNP location/dbSNP accession number	Genotypes	Genotype frequencies	Minor allele frequency	Deviations from HWE (*P*-value)
*rs43101491*	Upstream of the *GNAS *gene	58,215,520	GG	0.005	0.09 (G)	0.24
			GT	0.170		
			TT	0.825		
*rs43101493*	Upstream of the *GNAS *gene	58,214,963	AA	0.660	0.19 (G)	0.19
			AG	0.300		
			GG	0.040		
*rs43101486*	Upstream of the *GNAS *gene	58,203,762	AA	0.020	0.14 (A)	0.56
			AG	0.240		
			GG	0.740		
*rs43101485*	Upstream of the *GNAS *gene	58,203,464	CC	0.043	0.19 (C)	0.36
			CG	0.303		
			GG	0.654		
*rs41694646*	Intron 2 of the *GNAS *gene	58,183,623	CC	0.090	0.32 (C)	0.35
			CG	0.450		
			GG	0.460		
*rs41694656*	Intron 1 of the putative *NESP55 *gene	58,281,228	AA	0.747	0.14 (G)	0.25
			AG	0.229		
			GG	0.024		

Genotype frequency, minor allele frequency (MAF) and significance of deviation for Hardy-Weinberg equilibrium (HWE) based on *P*-values obtained from χ2-test results are given for all seven SNPs. dbSNP database accession numbers for each SNP were provided from GenBank [[53]; http://www.ncbi.nlm.nih.gov/projects/SNP] and the Build 4.0, release 60, of the *B. taurus *genome assembly in the ENSEMBL database http:/www.ensembl.org.

### An intronic SNP located between exon 2 and 3 of the bovine *GNAS *gene is associated with a range of production traits in cattle

The SNP genotype associations with performance traits for all six assayed SNPs are presented in Table 2 and 3--only significant genotype-phenotype associations are presented in these. Notably, one SNP (*rs41694646*), located in the intron separating the 2^nd ^and 3^rd ^exons of the *GNAS *gene (ENSEMBL database transcript ID ENSBTAT00000002746), displayed highly significant associations with a range of the cattle production traits assessed. The G-to-C allele substitution at this locus was associated with increases in milk yield (*P *≤ 0.001) and milk protein yield (*P *≤ 0.05) and decreases in milk fat and protein content (*P *≤ 0.05). No associations with milk fat yield were observed at this SNP locus. This SNP was also associated with carcass and growth/body conformation traits including culled cow carcass weight (*P *≤ 0.01), progeny carcass fat (*P *≤ 0.01), progeny carcass conformation (*P *≤ 0.01) and animal stature and body depth (both *P *≤ 0.001). However, no associations with rump angle and rump width were observed. The *rs41694646 *SNP also tended to be associated (*P *≤ 0.10) with cow angularity--a subjective measure of subcutaneous fat levels in live animals--with the G allele being associated with greater fat cover. The low pairwise *r^2 ^*values (≤ 0.030) observed for this SNP with all other genotyped *GNAS *domain SNPs in this study suggest the allele substitution effects at this locus are independent. Following adjustment for multiple testing, statistical associations (*q *≤ 0.05, where *q*-values represent the raw *P*-value corrected for multiple testing) between the *rs41694646 *SNP and three traits remained--milk yield, animal stature and body depth.

**Table 2:** Estimated allele substitution effect (standard error in parenthesis) between six SNPs in the bovine GNAS domain and milk performance, somatic cell count (SCC), calving and fertility traits

SNP	Allele substitution	Milk yield (kg)	Milk protein yield (kg)	Milk fat content^1 ^(%×100)	Milk protein content^1 ^(%×100)	SCC (units×100)	Direct calving difficulty^2^	Maternal calving difficulty^2^	Gestation (days)
*rs43101491*	G→T	8.65 (16.56)	0.25 (0.46)	-1.25 (1.25)	-0.10 (0.61)	0.97 (1.01)	-0.51** (0.19)	0.66** (0.23)	-0.12 (0.12)
*rs43101493*	A→G	4.24 (12.05)	0.16 (0.33)	0.85 (0.91)	0.13 (0.44)	-0.97 (0.73)	0.08 (0.14)	-0.19 (0.17)	0.05 (0.09)
*rs43101486*	A→G	-8.94 (13.58)	-0.05 (0.38)	0.11 (1.03)	0.37 (0.50)	1.36^†^ (0.81)	-0.16 (0.16)	0.26 (0.19)	-0.04 (0.10)
*rs43101485*	C→G	-2.61 (12.04)	-0.09 (0.34)	-0.85 (0.91)	-0.12 (0.44)	0.90 (0.72)	-0.12 (0.14)	0.19 (0.17)	-0.06 (0.09)
*rs41694646*	C→G	-34.63*** (10.48)	-0.63* (0.29)	1.98* (0.79)	0.94* (0.38)	-0.60 (0.63)	-0.44*** (0.12)	0.28^†^ (0.15)	-0.23** (0.08)
*rs41694656*	A→G	20.22 (14.04)	0.63 (0.39)	-0.79 (1.06)	-0.02 (0.52)	-2.37** 0.85	0.10 (0.17)	-0.29 (0.20)	0.01 (0.11)

Significance of difference from zero ^† ^= *P *≤ 0.10; * = *P *≤ 0.05; ** = *P *≤ 0.01; *** = *P *≤ 0.001.

^1 ^A value of 1, prior to multiplication by 100, equates to 1 percentage unit.

^2 ^Expressed in standard genetic deviation units.

Underlined values represent genotype-phenotype associations that have remained statistically significant (*q *≤ 0.05) after adjustment for multiple testing.

**Table 3:** Estimated allele substitution effects (standard error in parenthesis) of six SNPs in the bovine GNAS domain on growth performance and size

SNP	Allele substitution	Culled cow carcass weight (kg)	Progeny carcass conformation^1 ^(×100)	Progeny carcass fat^2 ^(×100)	Stature^3^ (×10)	Body depth^3 ^(×10)	Angularity^3 ^(×10)
*rs43101491*	G→T	1.17 (1.06)	0.07 (3.40)	-4.07 (2.96)	-0.93 (1.52)	-0.47 (1.42)	-1.90 (1.59)
*rs43101493*	A→G	-0.13 (0.76)	-2.03 (2.45)	2.91 (2.12)	0.10 (1.07)	0.31 1.01	0.51 (1.13)
*rs43101486*	A→G	-0.23 (0.83)	0.20 (2.69)	-1.64 (2.34)	-1.08 (1.20)	-0.28 (1.13)	-0.90 (1.27)
*rs43101485*	C→G	0.10 (0.75)	1.87 (2.42)	-2.70 (2.11)	0.01 (1.07)	-1.83 (1.00)	-0.29 (1.13)
*rs41694646*	C→G	-1.66** (0.65)	5.49** (2.11)	5.44** (1.82)	-2.97*** (0.92)	-2.88*** (0.87)	-1.87^†^ (0.97)
*rs41694656*	A→G	0.40 (0.88)	-1.65 (2.84)	0.34 (2.45)	-4.93 (1.30)	-1.09 (1.22)	-1.15 (1.39)

Significance of difference from zero ^† ^= *P *≤ 0.10; * = *P *≤ 0.05; ** = *P *≤ 0.01; *** = *P *≤ 0.001.

^1 ^Scale: 1 (poor)-15 (good)

^2 ^Scale 1 (low)-15 (high)

^3 ^Expressed in genetic standard deviation units.

Underlined values represent genotype-phenotype associations that have remained statistically significant (*q *≤ 0.05) after adjustment for multiple testing.

### SNPs within the *GNAS *locus are associated with reproduction traits in domestic cattle

In addition to associations with milk, carcass and body size traits, the *rs41694646 *SNP was also associated with a range of calving traits, including direct calving difficulty due to the size of offspring [*i.e*. a sire effect on calving difficulty] (*P *≤ 0.001) and gestation length (*P *≤ 0.01). Association between this SNP and direct calving difficulty remaining after adjustment for multiple testing (*q *≤ 0.05). This SNP also tended to be associated (*P *≤ 0.10) with maternal calving difficulty (*i.e*. due to the size of the maternal pelvic width) and perinatal mortality (a C-to-G substitution at this locus results in 0.17% decrease in the rate of perinatal mortality [standard error ± 0.10%])--no other SNP analysed in this study was associated with perinatal mortality. Significant associations with direct calving difficulty due to offspring size (*P *≤ 0.01) and maternal calving difficulty (*P *≤ 0.01) were also observed at the *rs43101491 *SNP, which is located 14.2 kb upstream of the *GNAS *gene; however these associations were no longer significant after adjustment for multiple-testing. No significant associations were observed between any of the above-listed traits and the remaining three SNPs located upstream of the *GNAS *gene (*i.e. rs43101491*, *rs43101486*, *rs43101485*).

### A non-synonymous SNP within the *NESP55 *cattle gene model is associated with somatic cell count

The single assayed SNP within the putative bovine *NESP55 *transcript (*rs41694656*) displayed no significant associations with any of the milk production, carcass, body conformation or calving traits analysed but it was associated (*P *≤ 0.01) with somatic cell count. However, association with somatic cell count no longer remained after adjustment for multiple testing. Sequence alignment indicates that this SNP lies within the first exon of putative maternally expressed *NESP55 *transcript (GenBank accession U77614.1); the *NESP55 *exon is situated ~89.9 kb upstream of the first G_S_α exon. The G-to-A nucleotide substitution at this locus results in a non-synonymous aspartic acid-to-asparagine amino acid substitution at amino acid position 192 in the *NESP55 *protein [51].

## Discussion

### SNPs within or proximal to the bovine *GNAS *gene and their association with cattle performance traits

Candidate gene studies, whereby DNA sequence polymorphisms are pre-selected for analysis based on their proximity to genes/loci known (or considered likely) to play a role in regulating a phenotype of interest, are considered as viable alternatives to genome-wide association (GWA) studies [25]. Such approaches are also regarded as having the added advantage of reducing both the number of false-positive genotype-phenotype associations (*i.e*. spurious associations detected due to chance) and false-negative genotype-phenotype associations (*i.e*. true associations that are erroneously rejected as a result of rigorous conventional statistical testing) commonly encountered during GWA studies [25,26,56,57].

In the present study, we have adopted a candidate gene approach by analysing DNA sequence variation in the bovine *GNAS *imprinting domain and a number of economically-important performance traits in cattle. In humans and mice, this domain consists of a number of reciprocally-imprinted and alternatively spliced genes and has been shown to have an important relationship with mammalian growth, development and disease in these species [12,16,22]. While the *GNAS *gene, which is integral to this domain, is preferentially maternally expressed in humans and mice, the current imprinting status of the bovine ortholog of *GNAS *has not yet been conclusively defined [44,45]. However, the degree of imprinting conservation between mammalian species suggests that this gene may also be epigenetically regulated in cattle [46,47]. Indeed, the bovine *NESP55 *gene which lies upstream of the bovine *GNAS *gene has previously been shown to be a maternally expressed imprinted gene by Khatib [51] and also by us in the current study, the evidence for which is discussed below.

Genotype-phenotype association analysis performed in the current study identified a number of statistically significant associations (*P *≤ 0.05) between SNPs distributed across the orthologous bovine *GNAS *domain and a number of cattle growth and development, milk, calving and health traits. To our knowledge, this is one of the first studies demonstrating that DNA sequence variation within the *GNAS *domain underlies quantitative phenotypic traits in cattle. These associations are most aptly demonstrated by the *rs41694646 *SNP located within the 2^nd ^intron of the bovine *GNAS *transcript. Notably, this SNP was associated with, (1) animal growth and development (as illustrated by associations with animal stature, body depth, culled cow carcass weight, progeny carcass conformation and progeny carcass fat deposition), (2) fertility (as illustrated by associations with gestation length), (3) milk production (as illustrated by associations with milk yield, milk protein yield, and milk fat and protein content), and (4) calving (as illustrated by associations with direct calving difficulty). In addition, the *rs43101491 *SNP located upstream of the *GNAS *gene was also associated with the two calving traits analysed here. It should be noted that significant associations between the *rs41694646 *SNP and animal stature, body depth, direct calving difficulty and milk yield remained after correction for multiple-testing (*q *≤ 0.05).

The phenotypic associations with SNP variation in the *GNAS *domain detected in this study are supported by genetic analysis of the *GNAS *domain in other mammalian species. Genetic defects within the human *GNAS *domain can cause similar physiological defects to those observed in knockout mice strains. Heterozygous mutations disrupting expression from the G_S_α promoter display symptoms characteristic of Albright hereditary osteodystrophy (AHO) including short stature, bracydactyly and neurological defects. In addition, maternal-specific inheritance of mutations in the *GNAS *gene can result in severe obesity and resistance to growth-regulating hormones. Alternatively, paternally-derived mutations in G_S_α promoter-generated transcripts do not lead to development of obesity or multi-hormonal resistance reflecting the paternal-silencing of this promoter [16,58].

In mice, paternally- and maternally-inherited null mutations associated within the *Gnas *gene cluster results in severe, albeit different, developmental and disease phenotypes. For example, heterozygous mice carrying maternally-derived knockout mutations within exon 2 of the *Gnas *gene (an exon common to all transcripts produced within the *Gnas *domain) display severe obesity, increased insulin sensitivity and increased perinatal mortality, while heterozygotes carrying a paternally-derived exon 2 knockout mutations (indicative of a loss of *Gnasxl *transcripts) also displayed increased perinatal mortality, greatly increased insulin sensitivity, hypermetabolism and reduced adiposity; homozygous individuals were embryonically lethal [59-61]. Furthermore, a missense mutation in *Gnas *exon 6 (referred to as the *Oed-Sml *mutation) causes post-natal growth retardation when paternally-inherited in heterozygotes; when maternally-inherited in heterozygotes this mutation result in marked subcutaneous oedema, obesity and increased neonatal mortality [62]. Collectively, these lines of evidence have led to suggestions that the XLα_S _isoform (encoded by the *Gnasxl*) functions to promote growth and increase lipid/fat content and metabolism during development, while the function of the G_S_α protein is to reduce growth, fat mass and metabolism during development [63]. Notably, the bovine *GNAS *SNPs assayed in this study showed similar phenotypic effects on growth and fat deposition. Although no significant associations with calf perinatal mortality was observed, there was an observed tendency for the *rs41694646 *SNP to be associated with this trait. Indeed, the observed association between the intronic *rs41694646 *SNP and the effect of the sire on calving difficulty may possibly be due to foetal growth effects on calving success.

The association of the *GNAS *imprinting locus with progeny carcass fat deposition in cattle is intriguing as it resonates with proposed theories for imprinting evolution of genes controlling non-shivering thermogenesis in animal species (such as cattle and other domestic animals) that display huddling behaviour [64,65]. Young mammals generate heat through non-shivering thermogenesis and conserve heat loss through social thermoregulation (huddling) [66-68]. In this regard, *GNAS *has been highlighted as a gene that produces both a maternally expressed promoter and a paternally expressed inhibitor of non-shivering thermogenesis, which is proposed to lead to an intragenomic conflict driving imprinting at the *GNAS *domain [64,65].

A recent molecular evolutionary analysis of 34 mammalian imprinted genes by our group found that *GNASxl *is only one of two imprinted transcripts/genes (the other being *OSBPL5*) which displays site-specific positive Darwinian selection consistent with the parental conflict theory for evolution of imprinted genes [69,70]. In addition, the biochemical evidence from studies of the *GNAS*-derived gene products in humans and mice is often cited as support for the parental conflict or kinship theory for the evolution of imprinting. This theory proposes that paternally-expressed imprinted genes can act in the developing offspring to recruit biological resources from the mother and hence promote offspring growth, whereas maternally-expressed genes act to restrict demand on maternal biological resources by inhibiting offspring growth [69]. Phenotypes associated with disruptions of the imprinted *GNAS *locus have been particularly highlighted as supporting evidence for the parental conflict theory for the evolution of imprinting [64,65,71]. While a similar scenario in cattle is tempting to speculate given the observed associations with growth at the bovine *GNAS *gene in this study, determining the relative phenotypic contributions from maternally- and paternally-derived alleles at the *rs41694646 *SNP was not feasible as the association analysis presented here was not conducted across a structured, multi-generational population with known ancestral relationships. However, based on the results presented in the current study the use of this SNP in future QTL mapping studies using structured, multi-generational populations may allow for some testing of the parental conflict theory in relation to the *GNAS *gene-derived transcripts in livestock.

### Genotype-phenotype association analysis between the a non-synonymous *NESP55 *SNP and somatic cell count

The *NESP55 *transcript is produced by the splicing of the first *NESP *exon and G_S_α exons 2-13; however, the entire *NESP55 *coding sequence is contained within its first exon with G_S_α exons 2-13 forming the 3'UTR of *NESP55 *transcripts. This transcript encodes the NESP55 protein which consists of 241 amino acids and is an acidic, soluble heat-stable chromogranin-like protein localised within large dense core granules of secretory cells [19,72,73]. Although *NESP55 *is expressed in a wide range of human and murine tissues (especially neuroendocrine tissue including adrenal medulla, pituitary and hypothalmus) its function remains unknown [16]. While gene knockout studies in mice have shown that elimination of maternally-derived *NESP55 *transcripts do not affect post-natal development, altered behavioural reactivity to novel environments were observed [74].

Regardless of the function of its encoded gene product, the observed association between the *rs41694656 *SNP and somatic cell count (SCC) is noteworthy. The SCC phenotype reflects the number of leukocytes per millilitre of milk and is an indicator of clinical and sub-clinical mastitis in cattle. Clinical mastitis is the most frequent cause of involuntary culling in North American dairy herds costing the US dairy industry an estimated $2 billion annually [75-78]. Both SCC and clinical mastitis are strongly positively correlated and therefore selection for reduced SCC, either through quantitative genetics or molecular genetics means, is expected to, on average, reduce the incidence of mastitis [79,80]. Recently, a microsatellite-based whole genome scan identified a BTA13 as harbouring a QTL for SCC in Danish Holstein cattle [81]. Notably, this QTL was located in the genomic region encompassing the *GNAS *domain. Given the significant association observed between the *NESP55 *SNP and SCC in the current study, it is possible that the *NESP55 *gene underlies this QTL for SCC or is linked to another genetic locus that is associated with this trait.

The G-to-A substitution at the *rs41694656 *SNP located within the *NESP55 *gene represents the only coding sequence polymorphism analysed in this study, and results in a non-synonymous aspartic acid to asparagine codon substitution at amino acid position 192 of the NESP55 protein. However, given the similar biochemical properties between these two amino acids (both are small polar amino acids) it seems unlikely that this SNP is causal for the SCC phenotypic effect observed in this study. Rather, it is more plausible to suggest that this SNP is associated through LD with causal regulatory mutations (or set of mutations) located proximal to, or within the bovine *NESP55 *gene that have not yet been identified.

It is important to note that while many QTL scans for performance traits (using multi-generation resource populations with known pedigree structure) have incorporated imprinting effects in their statistical model [82-84], this was not possible in the current study as the DNA samples used were derived from progeny-tested Holstein-Friesian sires. While the association of variation in a domain containing maternally expressed imprinted genes (*i.e. NESP55 *and possibly *GNAS *in cattle) with phenotypic data derived from progeny-tested sires seems somewhat incongruent, this can possibly be explained by the fact that the genetic merit for each of traits examined here is calculated from many descendents across multiple generations (with female intermediaries). Therefore, variation in sire-derived maternally expressed imprinted genes could still be associated with performance.

### Imprinting of the bovine *NESP55 *gene

To date, *NESP55 *is the only transcript within the *GNAS *domain complex which has been shown to be imprinted in cattle exhibiting a maternally expressed pattern of genomic imprinting [51]. Our results further indicate that *NESP55 *remains imprinted across many tissues of earlier stage foetuses (*e.g*. 8 week) than previously analysed (*i.e*. from 10-13 week old foetuses [51]) (Figure 1 and 2). In addition, we demonstrate that *NESP55 *is also epigenetically regulated as a maternally expressed imprinted gene in intestinal and placental samples (cotyledon) of the 8-10 day old foetal offspring (Figure 1 and 2).

## Conclusions

Overall, our results provide evidence that DNA sequence variation within the bovine *GNAS *imprinting domain is associated with a number of performance traits in domestic cattle. We also provide additional evidence (to earlier reports) indicating that the *NESP55 *gene in this domain is a maternally expressed imprinted gene in foetuses as early as 8 weeks old. This lends further support to the accumulating body of research indicating that imprinted genes (and the complex imprinting cluster domains they reside in) can harbour important quantitative trait loci for economically-relevant performance traits in domestic livestock species. These observations increase support for the inclusion of imprinted loci (and their associated DNA sequence polymorphisms) as molecular markers for future domestic animal improvement strategies.

## Methods

### Analysis of *NESP55 *expression

#### A. Foetal tissue samples collection

Foetal samples were collected from two abattoirs: (a) the Kildare Chilling Company (Kildare town, County Kildare, Ireland), and (b) Meadow Meats (Rathdowney, County Laois, Ireland). Upon collection the foetuses were immediately chilled on ice. After dissection, the obtained tissues were submerged in an appropriate volume of RNAlater^® ^solution (Applied Biosystems, Warrington, UK). In total, 10 foetuses ranging from 6-10 weeks old (based on the crown-rump length of the foetus) were collected. The tissues were kept at 4°C overnight, then the RNAlater^® ^solution was removed and tissues were frozen in liquid nitrogen and stored at -80°C.

#### B. RNA extraction from foetal tissue

Total RNA was extracted using RNAqueous^® ^Kit (Applied Biosystems, Warrington, UK) following the manufacturer's instructions. Approximately 75 mg of the frozen sample was removed and homogenised in 750 μl lysis buffer (supplied with the RNAqueous^® ^Kit) using a hand electric homogeniser. The lysate was then mixed with 750 μl of 64% v/v ethanol by inverting the tube several times. The lysate/ethanol mixture was applied to a filter cartridge supplied with the kit and centrifuged at 13,000×g for one minute. The flow-through was discarded and the cartridge was washed with a 700 μl of Wash Solution 1 and subsequently with 500 μl Wash Solution 2 and 3 (supplied with the RNAqueous^® ^Kit). Upon the addition of each appropriate solution the tubes were centrifuged at 13,000×g for one minute and the flow through was discarded. Total RNA was eluted twice with 50 μl of DNAse- and RNAse-free water, divided into aliquots, and quantified using a NanoDrop™ ND1000 spectrophotometer V 3.5.2 (Thermo Scientific Ltd., Wilmington, DE, USA). To validate RNA integrity two volumes of formaldehyde-based loading dye was added to each RNA sample and analysed on 1% agarose gel in 1× TBE after ethidium bromide staining.

#### C. cDNA synthesis

cDNA synthesis reactions were carried out using the QuantiTect Reverse Transcription Kit (Qiagen Ltd. Crawley, UK) following the manufacturer's instructions. Approximately 1 μg of total RNA in 12 μl RNAse-free water was mixed with 2 μl of genomic DNA (gDNA) Wipeout Buffer and incubated for 5 min at 42°C. The reaction mixture was then cooled on ice and 6 μl of reverse transcription master mix containing 1 μl of the Quantiscript^® ^reverse transcriptase enzyme (Qiagen Ltd. Crawley, UK), 1 μl of reverse transcription primer mix and 1× Quantiscript reverse transcription buffer was added to each RNA sample. The reaction was incubated at 42°C for 30 min and subsequently the reverse transcriptase enzyme was inactivated by placing the samples for 3 min in 95°C. cDNA samples were diluted 1:4 for further analysis.

#### D. gDNA PCR amplification, RT-PCR amplification and sequencing of *NESP55 *amplification products

PCR amplifications of gDNA for DNA sequence analysis were performed in 50 μl volume. RT-PCR amplifications were performed in 20 μl volume. PCR primers used for gDNA amplifications were located in the single *NESP55 *coding exon (forward primer sequence: 5'-AGTCCGAGACCGAATTCG-3'; reverse primer sequence: 5'-CATTAGCTGAGCCGGATGG-3'), while PCR primers for cDNA amplification were located in the *NESP55 *coding exon (forward primer sequence: 5'-AGTCCGAGACCGAATTCG-3') and exon 6 of the bovine *GNAS *gene (reverse primer sequence: 5'-CGTTGGAGCGCTCATAGCAG-3'), respectively. Each PCR reaction included 20 ng of DNA or cDNA; 0.4 μM of each primer; 1× Green GoTaq^® ^Flexi Buffer (Promega Ltd., Ireland); 0.25 mM of each dNTP (Sigma Aldrich Ltd., Ireland) and 0.4 Units of GoTaq^® ^Flexi DNA Polymerase (Promega Ltd., Ireland). MgCl_2 _solution was added to each PCR at a final concentration of 2.5 mM. For all PCR amplifications, an initial denaturation step of 5 min at 95°C was followed by 35 cycles of a 3-step amplification programme of 30 sec at 95°C for denaturation, 30 sec at 60°C for annealing and 1 min at 72°C for extension. The final extension step was performed after 35 cycles of the above process at 72°C for 5 minutes. All content of each PCR reaction was loaded on a 1% w/v agarose gel stained with ethidium bromide (Sigma Aldrich Ltd., Ireland) in 1× TBE buffer and visualised under UV light. All sequencing reactions were performed commercially by GATC Biotech Ltd. (Constance, Germany) using the primer sequences listed above and resulting DNA sequence traces were analysed using the LaserGene Package (DNASTAR, WI, USA).

### SNP genotype-phenotype association analysis

#### A. Bovine *GNAS *domain sequence analysis and SNP validation

Two alternatively spliced transcripts have described for the currently annotated bovine *GNAS *gene (ENSEMBL gene ID ENSBTAG00000017475) in the ENSEMBL database (http://www.ensembl.org, ENSEMBL release 60, November 2010). These are: (a) the *GNAS *transcript (ENSEMBL transcript ID ENSBTAT00000002746) which consists of eight translated exons and one untranslated exon and encodes a 253 amino acid protein, and (b) the *GNAS2_BOVIN *transcript (ENSEMBL transcript ID ENSBTAT00000023246) which consists of 16 translated exons encoding a 350 amino acid protein. The final eight translated exons of the *GNAS2_BOVIN *transcript represent the first eight translated exons of the *GNAS *transcript (Figure 3). A novel transcript (ENSEMBL transcript ID ENSBTAT00000002746) consisting of all alternatively spliced *GNAS *and *GNAS2_BOVIN *exons together with additional exon sequences has also been reported in the ENSEMBL database.

The methods used to validate DNA sequence polymorphisms for genotyping within the bovine *GNAS *domain have been discussed in detail elsewhere [52]. Briefly, high-fidelity PCR amplicons spanning putative SNPs within the bovine *GNAS *region on BTA13 as per Build 4.0 of the bovine genome in the ENSEMBL database were generated for a panel of 26 animals (comprising European *Bos taurus*, African *B. taurus *and Indian *B. indicus *animals) and sequenced bi-directionally (Macrogen Inc., Korea; http://www.macrogen.com). The MEGA 4.0 software package [85] was used to analyse all resulting DNA sequences and to confirm the presence of SNPs. In the current study, we used six validated *GNAS *domain SNPs (three transitions and three transversions) for high-throughput genotyping. One SNP (*rs41694646*) was located within the second intron of the *GNAS *gene (ENSEMBL gene ID ENSBTAG00000017475; ENSEMBL transcript ID ENSBTAT00000002746) while four SNPs (*rs43101491, rs43101493, rs43101486, rs43101485*) were located upstream of the bovine *GNAS *gene based on the currently annotated open reading frame (ORF) gene model of the *GNAS *gene.

The final SNP (*rs41694656*, a G-to-A nucleotide substitution) was located within the first exon of the bovine *NESP55 *gene. Previously, Khatib [51] used this SNP to detect imprinting of the bovine *NESP55 *gene, however given the major advances in bovine genomics resources since then, we confirmed the location of this SNP using a bioinformatics approach. At the time of analysis, the genomic DNA (gDNA) sequence of this gene was not fully annotated within *B. taurus *reference genome sequence. Instead, the location and gDNA sequence of *NESP55 *was identified via alignment of the complete *NESP55 *mRNA sequence (GenBank accession number U77614.1) with Build 4.0 of the bovine genome using the BLAT sequence alignment tool available through the UCSC genome browser http://genome.ucsc.edu. The amino acid sequence of the *NESP55 *complete transcript carrying the A allele at the *rs41694656 *SNP was produced using the 'Translate' option on the ExPASy proteomics server http://www.expasy.ch. Alignment of the resulting amino acid sequences revealed that the G-to-A nucleotide substitution at the *rs41694656 *SNP causes an aspartic acid (codon GAC) to asparagine (codon AAC) at amino acid position 192 of the *NESP55 *protein, thus confirming the findings of Khatib [51].

#### B. DNA samples, DNA extraction, high-throughput SNP genotyping and SNP data filtering

Genomic DNA from 914 progeny-tested Irish Holstein-Friesian sires was purified using a Maxwell™ 16 automated apparatus (Promega Corp., Madison, WI, USA) as per manufacturer's instructions. These sires have been used to produce progeny in Ireland (via artificial insemination) and were representative of the commercial germplasm used in Irish dairy herds in past years. Genotyping for all six *GNAS *SNPs was performed on all 914 sires together with an additional 25 independently-extracted, duplicate samples that were included for genotype quality control purposes. All SNP genotyping was performed commercially by Sequenom Inc. (San Diego, CA, USA; http://www.sequenom.com using their proprietary MassARRAY iPLEX™ Gold genotyping platform. This SNP genotyping platform discriminates between SNP alleles using single base primer extension technology after which primer extension products are analysed using matrix-assisted laser desorption ionisation time-of-flight (MALDI-TOF) mass spectroscopy http://www.sequenom.com/iplex. Furthermore, this SNP genotyping platform has been validated by us in a previous study [86].

Genotype quality control and data filtering were performed on all data prior to association analyses. This involved the use of an iterative algorithm to remove SNPs and individuals that yielded poor genotype call rates. Firstly, SNPs with a genotype call rate < 75% across all 914 individuals were removed, followed by the removal of individuals with genotype call rates of < 85% across all remaining SNPs--this resulted in the removal of 21 sires and no SNPs from the study. Secondly, SNPs that yielded genotypes in < 90% of all remaining 893 individuals were discarded followed by the removal individuals that failed to yield a genotype for < 90% of all remaining SNPs--this resulted in the removal of a further 45 sires from the study, while no SNPs were discarded after the second filtering process.

After data filtering, genotypic data for all six SNPs and 848 progeny-tested sires with an average co-ancestry of 2.2% remained. The SNP genotype concordance rate between technical replicate for these SNPs was 100%. Summary statistics for each SNP (including allele and genotype frequencies) and phenotype association analyses were performed using this edited dataset. *D*' and *r*^2 ^estimates of linkage disequilibrium (LD) [54,55] between every pairwise combination of segregating SNPs across the *GNAS *domain were also generated from this edited dataset using the HAPLOVIEW software package [87].

#### C. Phenotypic data and association analyses

A range of phenotypic traits were analysed in this study and were categorised into six broad categories: (1) *milk production traits *[milk yield, fat yield, protein yield and milk fat and protein concentration], (2) *udder health *[somatic cell count, SCC], (3) *carcass traits *[culled cow carcass weight, progeny carcass weight, progeny carcass conformation score and progeny subcutaneous carcass fat level], (4) animal size in live animals [animal stature, body depth, chest width, rump angle and rump width]; (5) *subjectively assessed subcutaneous fat level on live animals *[angularity and body condition score], and (6) *calving traits *[calving difficulty (both direct and maternal calving difficulty), gestation length and perinatal mortality]. All phenotypic data were kindly supplied by the Irish Cattle Breeding Federation http://www.icbf.com and a detailed description of all traits is provided in Additional File 2.

The phenotypes used in this study are sire genetic merit based not on data on the sires themselves but on the performance of their female progeny across multiple generations. Using known relationships among animals, performance records on relatives are used to estimate the genetic merit of an animal (*i.e*. a sire). Systematic environmental effects on the progeny are adjusted for and the random non-genetic variation associated with the progeny's phenotypes is minimised, thus facilitating a more accurate measure of genetic merit. This increased study power is particularly beneficial for low heritability traits where the proportion of phenotypic variance attributable to additive genetic differences is low. The disadvantage of such a study design is that the performance traits included for analysis are limited to those routinely measured on progeny. The average number of progeny per sire analysed here was 842 daughter-parity records. When coupled with the mixed model methodology used and the de-regression of the predicted transmitting ability (PTA), this implies that the associations reported herein are independent of pedigree structure.

Sire PTA (*i.e*. the average genetic merit for a given trait that an animal transmits to its offspring) was the dependent variable for all traits with the exception of the milk production traits, including somatic cell count, which were daughter yield deviations (DYDs, the average of a sire's daughters' performance) expressed on a PTA scale. Models used in genetic evaluations in Ireland, as well as variance components, have been summarised in detail previously [88]. DYDs for 305-day milk, fat and protein yield as well as geometric mean SCC (log_e _somatic cell count) are estimated in Ireland using a repeatability animal model across the first five lactations. PTAs for calving interval and survival are estimated using a multi-trait animal model, including data from the first three lactations. PTAs for milk yield are used to adjust PTA for survival for differences in genetic merit of milk yield; hence, this survival trait is functional survival. PTAs for cow carcass weight, progeny carcass weight, progeny carcass fat score (scale 1 to 15; Hickey *et al. *[89]) and progeny carcass conformation score (scale 1 to 15; Hickey *et al. *[89]), measured at slaughter, are estimated in a multi-trait animal model. Cows slaughtered between 875 and 4,000 days of age are included in the evaluation of cow weight while male progeny slaughtered between 300 and 1,200 days of age and female progeny slaughtered between 300 and 875 days of age are included in the evaluation of the remaining three carcass traits.

Genetic evaluations for calving ease are undertaken using a bivariate animal-dam model so that PTAs for direct and maternal calving ease are both generated. In the bivariate model the breeding-goal trait (*i.e*. the phenotypic trait wished to be improved genetically) is calving ease scored by commercial Irish farmers and the predictor trait is calving ease scored prior to 2002 in progeny test and pedigree herds. A similar approach is used to estimate breeding values for gestation length and perinatal mortality with the exception that an animal model is used. Direct calving difficulty refers to the additive genetic effect of the genotype of the calf (*e.g*. size of the calf) while maternal calving difficulty refers to the additive genetic effect of the genotype of the calf's dam (*e.g*. pelvic weight of the dam). Perinatal mortality is a dichotomous variable scored by farmers as calf dead at birth or within 24 hours [90].

Genetic evaluations for angularity and body condition score are undertaken as part of a joint evaluation in the UK and Ireland. The estimated breeding values (EBVs) are standardised to the mean and standard deviation of the base population. Both angularity and BCS are subjective measures of the subcutaneous fat levels of the live animal. All PTAs were deregressed using the procedure outlined by Berry and colleagues [91].

Only sires with a reliability, less parental contribution, of > 60% were retained for inclusion in the association analysis. A total of 742 sires fulfilled these criteria for inclusion in the analysis of milk, fat and protein yield as well as milk fat and protein concentration. 501 sires were included in the association analysis with calving interval, while 477 sires were included for association analysis with calf survival. The number of sires with a reliability of > 60% for the carcass traits was 446 and the number of sires with a reliability of > 60% for angularity and body condition score varied and was 521 and 504, respectively. The main advantage of using high reliability sire PTAs generated from progeny performance is the increased accuracy of the phenotype compared to actual phenotypes of individual animals. This is particularly true for low heritability traits where the accuracy of the genetic merit of an animal based on a single measure is low (*i.e*. square root of the heritability). The use of highly accurate phenotypes in association analyses is vital to obtain accurate estimates of associations.

The association between each SNP and performance was quantified using weighted mixed linear models in ASREML [92] with individual included as a random effect, and average expected relationships among individuals accounted for through the numerator relationship matrix. Year of birth (divided into five-yearly intervals) and percent Holstein of the individual bull were included as fixed effects in the model. In all instances the dependent variable was de-regressed PTA or DYD, weighted by their respective reliability less the parental contribution. Genotype was included in the analysis as a continuous variable coded as the number of copies of a given allele.

Regression on individual SNPs were initially undertaken to identify spatial patterns of SNPs associated with performance. Because of the covariances between SNPs (*i.e*. linkage disequilibrium), and between phenotypes, traditional multiple testing adjustments that assume independence among the regressors (*e.g*. Bonferroni permutation) were not appropriate. Spectral decomposition of the square root of the pair-wise linkage disequilibrium between the SNPs was used to determine the effective number of variables (*i.e*. SNPs); an effective number of 4.22 SNPs were identified. Furthermore, the phenotypes were grouped into six categories as described previously. Adjustment [93] for multiple testing was therefore undertaken assuming a total of therefore 25.37 effective independent tests (*i.e*. 4.22 effective SNPs times six groups of traits).

All research was conducted in accordance with the ethical guidelines and procedures of the UCC and UCD Animal Ethics Committees.

## Authors' contributions

KMS, DAM and EWB performed laboratory work associated with this study. KMS validated the SNPs genotyped, analysed genotypic data and investigated the imprinting status of the *NESP55 *gene. DAM, EWB provided assistance with SNP validation, prepared DNA samples for genotyping and performed data analysis. DJH and MPM extracted DNA from semen samples and prepared DNA samples for genotyping. DPB collected phenotypic data for the animals used in this study and performed statistical analyses of the genotypic and phenotypic data. RDE contributed to the collection and statistical analysis of the phenotypic data used in this study and provided valuable comments and discussion towards the final manuscript. DEM and CS conceived the study and participated in its design and coordination. KMS, DAM, DPB, DEM and CS wrote and edited the manuscript. All authors read and approved the final manuscript. All research was conducted in accordance with the ethical guidelines and procedures of the UCC and UCD Animal Ethics Committees.

## Supplementary Material

Additional file 1 ***GNAS *domain pairwise SNP linkage disequilibrium (LD) values**. This Microsoft Excel file contains *D*' and *r*^2 ^measures of LD for each *GNAS *domain pairwise SNP combination. Click here for file

Additional file 2 **Descriptions of the performance traits assessed in the present study**. This Microsoft Word file contains detailed information for each of the phenotypic trait analysed as provided by the Irish Cattle Breeding Federation (ICBF) http://www.icbf.com. Click here for file
