# Supplementary material for: DNA sequence polymorphisms within the bovine guanine nucleotide-binding protein Gs subunit alpha (Gsα)-encoding (GNAS) genomic imprinting domain are associated with performance traits
Source: BMC Genet. 2011 Jan 7;12:4. doi: 10.1186/1471-2156-12-4 (PMC3025900; doi:10.1186/1471-2156-12-4)
Supplement: Additional file 2 — Descriptions of the performance traits assessed in the present study. This Microsoft Word file contains detailed information for each of the phenotypic trait analysed as provided by the Irish Cattle Breeding Federation (ICBF) http://www.icbf.com. [file 1471-2156-12-4-S2.DOC]

**Additional File 2:** Details of the phenotypes analysed in this study (all data provided by the Irish Cattle Breeding Federation [ICBF], http://www.icbf.com)

| **Trait information** | **Trait definition** |
| --- | --- |
| **Milk production traits measured on milk recording herds** | |
| Milk yield | The quantity of milk produced each year by a cow up to 305 days in milk for a given parity. Measured in kilograms (kgs). Parities 1 to 5 are used in Irish Cattle Breeding Federation (ICBF) genetic evaluations. Cow must have a known sire. Minimum criteria of 1500 kg 305 days milk yield. Published in dairy cattle as a predicted transmitting ability of a sire or dam. |
| Milk fat yield | The quantity of fat contained within milk. Measure in kgs up to 305 days in milk for a given parity. Parities 1 to 5 are used in ICBF genetic evaluations. Cow must have a known sire. Minimum criteria of 40 kg 305 days fat yield. Published in dairy cattle as a predicted transmitting ability of a sire or dam. |
| Milk protein yield | The quantity of protein contained within milk. Measure in kgs up to 305 days in milk for a given parity. Parities 1 to 5 are used in ICBF genetic evaluations. Cow must have a known sire. Minimum criteria of 40 kg 305 days protein yield. Published in dairy cattle as a predicted transmitting ability of a sire or dam. |
| Milk fat percentage | The total quantity of milk fat divided by the total quantity of milk produced each year by a cow. Measured as a percentage of the total milk yield. Published in dairy cattle as a predicted transmitting ability of a sire or dam |
| Milk protein percentage | The total quantity of milk protein divided by the total quantity of milk produced each year by a cow. Measured as a percentage of the total milk yield. Published in dairy cattle as a predicted transmitting ability of a sire or dam |
| **Udder health traits measured on milk recording herds** | |
| Somatic cell count (SCC) | A measure of the number of somatic cells (i.e. white blood cells/leukocytes) contained within the milk produced by a cow. The number of somatic cells increases in the milk in response to bacterial infections of the udder, usually mastitis (caused by the bacterium *S. aureus*). SCC is therefore regarded as an indirect measure of milk quality and the health of the cow. SCC is measures at the number of cells per mil of milk. Trait evaluated is somatic cell score which is calculated as LOGe(SCC). Cow must have a known sire. Published in dairy and beef cattle as a predicted transmitting ability of a sire or dam |
| **Carcass traits measured in Irish slaughter houses** | |
| Culled cow carcass weight | Carcass weight refers to the cold weight of the carcass taken within 2 hours of slaughter after being bled and eviscerated and after removal of skin, removal of external genitalia, the limbs at the carpus and tarsus, head, tail, kidneys and kidney fats and the udder. The (culled) cow carcass weight refers to the carcass weight of a dairy or beef cow slaughtered for meat at the end of her productive life. Measured in kgs on cows aged between 875 and 4000 days of age. Cow must have a known sire. Published in dairy and beef cattle as a predicted transmitting ability of a sire or dam |
| Progeny carcass weight | The carcass weight of a sire’s offspring/progeny. Measured on males from 300 – 1200 days and females from 300 – 875 days of age. (Females which have not produced a calf). Animal must have a known sire. Published in dairy and beef cattle as a predicted transmitting ability of a sire or dam |
| Subcutaneous progeny carcass fat level | The quantity of fat on the carcass of the slaughtered animal. Measured in kgs. Measured through the mechanical grading of cattle carcasses in Ireland (since 2003) for conformation and fat using the VBS2000 technology (EplusV GmbH, Germany). Subsequent phenotypes for genetic evaluations are created on a scale of 1 (leanest) to 15 (fattest). In the current study, values have been multiplied by 100 for convenience. Animal must have a known sire. Published in dairy and beef cattle as a predicted transmitting ability of a sire or dam |
| Progeny carcass conformation score | The thickness of muscle on the carcass of the slaughtered animal. Measured through the mechanical grading of cattle carcasses in Ireland (since 2003) for conformation and fat using the VBS2000 technology (EplusV GmbH, Germany). Subsequent phenotypes for genetic evaluations are created on a scale of 1 (poor conformation) to 15 (good conformation) after transformation from the European grading standard (SEUROP scale). In the current study, values have been multiplied by 100 for convenience. Published in dairy and beef cattle as a predicted transmitting ability of a sire or dam |
| **Linear assessment related traits recorded by ICBF linear scorers** | |
| (Animal) Stature | A measure of the height of the animal from the top of the spine in between the hips to the ground. Measured on a scale from 1 to 9. A score of 1 indicates a short animal, a score of 3 indicates an intermediate animal and a score of 9 indicates a tall animal. In the current study, the SNP allelic substitution effects are measured as standard deviation units (SDUs) – the score from the population/herd mean using the population/herd standard deviation of the trait as a unit of measure. Published in dairy cattle as a predicted transmitting ability of a sire or dam |
| Chest width | A measure of the chest width of the animal taken from inside surface of the chest between the top of the animal’s front legs. Phenotypic measures 1-3 = narrow chest; 4-6 = intermediate chest; 7-9 = wide chest. Intermediate values are regarded as favourable. In the current study, the SNP allelic substitution effects are measured as standard deviation units (SDUs). Published in dairy cattle as a predicted transmitting ability of a sire or dam |
| Body depth | A measure of the depth of the body taken as the distance from the top of the spine and the bottom of the last rib, which is the deepest section of the animal. This measure is independent of animal stature. Phenotypic measures 1-3 = shallow body depth; 4-6 = intermediate body depth; 7-9 = deep body depth. Intermediate values are regarded as favourable In the current study, the SNP allelic substitution effects are measured as standard deviation units (SDUs). Published in dairy cattle as a predicted transmitting ability of a sire or dam |
| Rump angle | A measure of the angle of the rump structure from the animal’s hips (hooks) to the back legs (pins). Phenotypic measures: 1 = high pins (pins are 4 cm higher than hips); 3 = level (pins and hips are level); 5 = intermediate (pins are 4 cm lower than the hips); 9 = extreme slope (pins are 12 cm lower than the hips). Level to intermediate values are regarded as favourable. In the current study, the SNP allelic substitution effects are measured as standard deviation units (SDUs). Published in dairy cattle as a predicted transmitting ability of a sire or dam |
| Rump width | A measure of the distance between the most posterior point of the pin bones. Phenotypic measures: 1-3 = narrow rump width; 4-6 = intermediate rump width; 7-9 = wide rump width. Intermediate values are regarded as favourable In the current study, the SNP allelic substitution effects are measured as standard deviation units (SDUs). Published in dairy cattle as a predicted transmitting ability of a sire or dam |
| **Subjectively assessed measure of subcutaneous of fat on a live animal** | |
| Angularity | Defined as a visual measure angle and openness of the ribs, combined with a measure of bone quality. Regarded as a subjectively assessed measure of subcutaneous of fat on a live animal. Phenotypic measures: 1-3 = not angular (high fat deposition); 4-6 = intermediate angularity; 7-9 = extremely angular. Published in dairy cattle as a predicted transmitting ability of a sire or dam |
| Body condition score | A scoring procedure which describes the state of fleshing of the animal. Published in dairy cattle as a predicted transmitting ability of a sire or dam |
| **Calving traits measured on Irish dairy and beef herds** | |
| Direct calving difficulty | The difficulty in calving attributed to the direct genes of the calf born. Phenotypic (numerical) scores for this trait range from 1= easy, unassisted calving, 2 = Some assistance, 3 = considerable difficulty to 4 = Veterinary assistance or Caesarean section. Animal must have a known sire. Published in dairy and beef cattle as a predicted transmitting ability of a sire or dam. |
| Maternal calving difficulty | The difficulty in calving attributed to the maternal genes of the dam. Related to the pelvic structure of the dam. Phenotypic (numerical) scores for this trait range from 1= easy, unassisted calving, 2 = Some assistance, 3 = considerable difficulty to 4 = Veterinary assistance or Caesarean section. Cow and calf must have a known sire. Published in dairy and beef cattle as a predicted transmitting ability of a sire or dam. |
| Calf (perinatal) mortality | Calf mortality shortly before, during and within 24 or 48 h after parturition. This trait is associated with a number of genetic and non-genetic factors. In the current study, allelic substitution effects associated with this trait are measured as a percentage of the total number of perinatal mortalities recorded for offspring of the progeny-tested sires. |
| Calf survival | Dead or alive at 28 days, This trait is combined with calf (perinatal) mortality in a genetic evaluation for mortality. Published in dairy and beef cattle as a predicted transmitting ability of a sire or dam |
| **Fertility traits measured on Irish dairy and beef herds** | |
| Gestation length | The number of days between known conception date and subsequent calving date. Calf must have a known sire. Published in dairy and beef cattle as a predicted transmitting ability of a sire or dam |
| Calving interval | The time in days between consecutive calvings for a cow. Parities 1 to 3 included in a joint evaluation with cow survival. Cow must have a known sire. Published in dairy and beef cattle as a predicted transmitting ability of a sire or dam |
